# Revised eutherian gene collections

**DOI:** 10.1186/s12863-022-01071-9

**Published:** 2022-07-23

**Authors:** Marko Premzl

**Affiliations:** 1 The Australian National University Alumni, 4 Kninski trg Sq., Zagreb, Croatia; 2 https://www.ncbi.nlm.nih.gov/myncbi/mpremzl/cv/130205/

**Keywords:** Gene data set, Comparative genomics, Eutheria, RRID:SCR_014401

## Abstract

**Objectives:**

The most recent research projects in scientific field of eutherian comparative genomics included intentions to sequence every extant eutherian species genome in foreseeable future, so that future revisions and updates of eutherian gene data sets were expected.

**Data description:**

Using 35 public eutherian reference genomic sequence assemblies and free available software, the eutherian comparative genomic analysis protocol RRID:SCR_014401 was published as guidance against potential genomic sequence errors. The protocol curated 14 eutherian third-party data gene data sets, including, in aggregate, 2615 complete coding sequences that were deposited in European Nucleotide Archive. The published eutherian gene collections were used in revisions and updates of eutherian gene data set classifications and nomenclatures that included gene annotations, phylogenetic analyses and protein molecular evolution analyses.

## Objective

The most recent research projects in scientific field of eutherian comparative genomics included intentions to sequence every extant eutherian species genome in foreseeable future, so that future revisions and updates of eutherian gene data sets were expected [1–13]. For example, the human protein coding gene census remained unfinished: contemporary estimates included about 20,000–21,000 protein coding genes in human genome [14–27]. In addition, the proven utility of public eutherian reference genomic sequences could become compromised by potential genomic sequence errors, including analytical and bioinformatical errors, as well as Sanger DNA sequencing method errors [28–33].

## Data description

Using public eutherian reference genomic sequence assemblies and free available software, the eutherian comparative genomic analysis protocol was published as guidance against potential genomic sequence errors [34–49]. The protocol included 3 major processing steps that were integrated into one framework of eutherian gene data set descriptions: gene annotations, phylogenetic analysis and protein molecular evolution analysis. The protocol published 3 original genomics and protein molecular evolution tests. First, the test of reliability of public eutherian genomic sequences used genomic sequence redundancies of public eutherian reference genomic sequence assemblies. Second, the test of contiguity of public eutherian genomic sequences used multiple pairwise genomic sequence alignments. Third, the test of protein molecular evolution used relative synonymous codon usage statistics. The protocol was made available on Protocol Exchange [44].

In aggregate, the eutherian comparative genomic analysis protocol curated 14 eutherian gene data sets implicated in major physiological and pathological processes, including 2615 published complete coding sequences that were made available in public biological databases as third-party data gene data sets [50–63] (Table 1). The curated gene data sets were deposited in European Nucleotide Archive [7–9, 12, 13] in FASTA nucleotide sequence format. The published eutherian gene collections were used in revisions and updates of eutherian gene data set classifications and nomenclatures.

**Table 1:** Overview of eutherian third-party data gene data sets

Label	Name of data file/data set	File types (file extension)	Data repository and identifier (DOI or accession number)
Data set 1	Interferon-γ-inducible GTPase genes (FR734011-FR734074)	FASTA (.fas)	European Nucleotide Archive (https://identifiers.org/ena.embl:FR734011) [50]
Data set 2	Adenohypophysis cystine-knot genes (HF564658-HF564785)	FASTA (.fas)	European Nucleotide Archive (https://identifiers.org/ena.embl:HF564658) [51]
Data set 3	Macrophage migration inhibitory factor genes (HF564786-HF564815)	FASTA (.fas)	European Nucleotide Archive (https://identifiers.org/ena.embl:HF564786) [52]
Data set 4	Ribonuclease A genes (HG328835-HG329089)	FASTA (.fas)	European Nucleotide Archive (https://identifiers.org/ena.embl:HG328835) [53]
Data set 5	Mas-related G protein-coupled receptor genes (HG426065-HG426183)	FASTA (.fas)	European Nucleotide Archive (https://identifiers.org/ena.embl:HG426065) [54]
Data set 6	Lysozyme genes (HG931734-HG931849)	FASTA (.fas)	European Nucleotide Archive (https://identifiers.org/ena.embl:HG931734) [55]
Data set 7	Growth hormone genes (LM644135-LM644234)	FASTA (.fas)	European Nucleotide Archive (https://identifiers.org/ena.embl:LM644135) [56]
Data set 8	Tumor necrosis factor ligand genes (LN874312-LN874522)	FASTA (.fas)	European Nucleotide Archive (https://identifiers.org/ena.embl:LN874312) [57]
Data set 9	Globin genes (LT548096-LT548244)	FASTA (.fas)	European Nucleotide Archive (https://identifiers.org/ena.embl:LT548096) [58]
Data set 10	Kallikrein genes (LT631550-LT631670)	FASTA (.fas)	European Nucleotide Archive (https://identifiers.org/ena.embl:LT631550) [59]
Data set 11	Adiponectin genes (LT962964-LT963174)	FASTA (.fas)	European Nucleotide Archive (https://identifiers.org/ena.embl:LT962964) [60]
Data set 12	Connexin genes (LT990249-LT990597)	FASTA (.fas)	European Nucleotide Archive (https://identifiers.org/ena.embl:LT990249) [61]
Data set 13	Fibroblast growth factor genes (LR130242-LR130508)	FASTA (.fas)	European Nucleotide Archive (https://identifiers.org/ena.embl:LR130242) [62]
Data set 14	Interferon genes (LR760818-LR761312)	FASTA (.fas)	European Nucleotide Archive (https://identifiers.org/ena.embl:LR760818) [63]

## Limitations

The revisions and updates of eutherian gene data sets were contingent on primary Sanger DNA sequencing information deposited in National Center for Biotechnology Information NCBI Trace Archive [12, 13, 46, 64–66]. For example, the positive correlation was calculated between genomic sequence redundancies of 35 public eutherian reference genomic sequence assemblies respectively and curated complete coding sequence numbers.

## Data Availability

The data described in present Data note could be freely and openly accessed in European Nucleotide Archive under accessions: FR734011-FR734074, HF564658-HF564785, HF564786-HF564815, HG328835-HG329089, HG426065-HG426183, HG931734-HG931849, LM644135-LM644234, LN874312-LN874522, LT548096-LT548244, LT631550-LT631670, LT962964-LT963174, LT990249-LT990597, LR130242-LR130508 and LR760818-LR761312. Please, see Table 1 and references [50–63] for details and URLs.
